# Cases of Amenorrhœa, Treated with the Ammoniated Tincture of Guaiacum

**Published:** 1826-12

**Authors:** George Jewel

**Affiliations:** Surgeon to the Middlesex Infirmary.


### AMENORRHŒA.

*Cases of Amenorrhœa, treated with the Ammoniated Tincture of Guaiacum.* By George Jewel, Surgeon to the Middlesex Infirmary.

There are no cases, when protracted, more obstinate, and none which occasion greater apprehensions on the part of the patient and her friends, than those of amenorrhœa; and, notwithstanding the usual and necessarily diversified means of cure which are commonly employed, they too frequently terminate, after a long series of varied and distressing symptoms, in the death of the patient, either from actual disease of some portion of the uterine system, or other more distant but important organ. I have been induced to publish a statement of the following cases, in the treatment of which I have been successful, as the medicine employed, (the ammoniated tincture of guaiacum,) is not, in my opinion, appreciated so highly as its properties seem to demand; a circumstance probably arising from its not having received so much attention, as an emenagogue, in this country as in some others. In the administration of this medicine, it is but justice to state that I have experienced that which in common we experience from all—occasional disappointment: at the same time, I conceive there is no medicine whose effects are more certain, provided the catamenial suppression does not exist as the consequence of any organic disease. Much, however, will depend upon the existing state of the system; for, as the qualities of the medicine are highly stimulating, it is of importance that the circulation should previously be brought down rather below the natural standard.

Catharine A—, a widow, ætatis thirty, was admitted a patient at the Middlesex Infirmary, Great Pulteney-street, on the 24th of June, having had a suppression of the catamenia during fourteen months. Her general leucophlegmatic appearance indicated great torpor of the whole system, and considerable derangement of the digestive functions. Appetite impaired; furred tongue; alvine evacuations irregular; respiration hurried, and palpitation of the heart upon the slightest exertion. Her legs and feet were œdematous, and she complained of occasional headache. The pulse, notwithstanding these indications of debility, was by no means feeble, but, on the contrary, rather full, accelerated, and quick. She was ordered to refrain from all spirituous and malt liquors, to live on a spare vegetable diet, and take as much exercise as the system could sustain without fatigue. A tea-spoonful of the Tinctura Guaiaci Ammoniata was ordered to be taken morning and evening, in a tea-cupful of sweetened milk; and the bowels to be kept open by a laxative pill of Colocynth and Gamboge.

After a fortnight's perseverance in this plan, the patient having experienced little or no relief, another tea-spoonful was taken in the day; and it was strongly impressed upon her mind that she was not to anticipate a cure without perseverance.

At the expiration of six weeks, a most extraordinary alteration was produced in her general health: her cheeks had now a healthy colour; her appetite had returned; the swelling of her legs had subsided, and the catamenial secretion had reappeared. She was discharged in perfect health on the 27th of August.

It is worthy of remark, that this case had not only been considered one of peculiar obstinacy, but almost hopeless, as she had consulted a variety of practitioners, and had taken steel to an immense extent. On calculating the quantity of the ammoniated tincture of guaiacum which this patient had taken from the commencement to the termination of her illness, I find that it must have exceeded twelve ounces.

II.—On the 14th of October, 1825, I was requested to visit Miss C—, a young lady, nineteen years of age, residing a short distance from town. In this case the catamenia had been suppressed four periods; the system appeared to have suffered materially; and she had been troubled a good deal with cough and occasional expectoration, symptoms which were viewed as presaging a fatal termination, there being an hereditary phthisis in the family. The functions of digestion were much impaired, with anorexia, and extreme languor of the circulation.

After evacuating the primæ viæ with rhubarb and the submuriate of mercury, repeated at intervals of two or three days, pills containing steel and myrrh were ordered, which she regularly took during five weeks, without the slightest visible amendment in the general health, and without restoring the uterine function. I then prescribed the Ammoniated Tincture of Guaiacum, in the usual manner; which medicine she steadily persevered in, with an occasional purgative, for about six weeks, when the catamenial secretion reappeared: the system immediately recruited its lost vigour, the cough gradually subsided, and she has ever since enjoyed perfect health and strength.

III.—A girl, ætatis fifteen, the daughter of a poor woman residing in Wellington Mews, Windmill-street, was brought as a patient to the Middlesex Infirmary, on the 16th of May, 1826. She had never menstruated, and complained of severe headache, resulting from a determination to the cerebral vessels, pains in the limbs, and constipation of the bowels. She was bled to the extent of eight ounces, and ordered to live on spare diet; to use regular exercise; and to take pills of the Extract of Colocynth and Calomel pro re nata.

After this plan had succeeded in reducing the system to a proper standard, the Pil. Ferri cum Myrrh. was ordered, which she continued to take some time, but, as in the former case, without producing the effect desired. She then commenced the use of the Ammoniated Tincture of Guaiacum, which in about three weeks completely succeeded in stimulating the uterine system to its proper and healthy function.*

---

### NEURALGIA.

* We have employed the Ammoniated Tincture of Guaiacum frequently, during the last two years, in cases of amenorrhœa unattended with inflammatory symptoms, and in general with very satisfactory results.—EDITOR.

